# Low autopsy acceptance after stillbirth in a disadvantaged French district: a mixed methods study

**DOI:** 10.1186/s12884-019-2261-3

**Published:** 2019-04-05

**Authors:** Priscille Sauvegrain, Marion Carayol, Aurélie Piedvache, Esther Guéry, Martine Bucourt, Jennifer Zeitlin

**Affiliations:** 1 Inserm UMR 1153, Obstetrical, Perinatal and Pediatric Epidemiology Research Team (Epopé), Center for Epidemiology and Statistics Sorbonne Paris Cité, DHU Risks in Pregnancy, Paris Descartes University, Maternité de Port Royal, 53, av. de l’Observatoire, 75014 Paris, France; 2 Department of Obstetrics and Gynecology, Pitié-Salpêtrière Hospital, AP-HP, Paris, France; 3 Maternal and Infant Protection Service, Department of Families and Early Childhood, Paris, France; 4 Fetopathology Unit, Jean Verdier Hospital, AP-HP, Bondy, France

## Introduction

Stillbirth continues to be a major public health concern in high income countries as highlighted by recent international collaborations which have shown the need for more research and policy for prevention and support for parents experiencing stillbirth [1]. Furthermore, risks of stillbirth are marked by strong social and geographical inequalities [2]. Stillbirth rates in disadvantaged communities can be double those in more affluent areas [3, 4]. In general, stillbirth rates are higher for women with lower educational levels, income and also within migrant communities. Including women with these risk factors in research on prevention and bereavement care is therefore essential.

One of the obstacles for developing effective policies are limits to current knowledge of the causes of stillbirth as up to half of stillbirths remain unexplained after review of clinical data. Autopsies are accepted as the gold standard for determining cause of death and the proportion of cases with no identified cause is lower when autopsy results are available [5–8]. In addition to informing prevention public policies, autopsies make it possible to counsel parents for future pregnancies and provide valuable information that can help in the grieving process [9]. However, many families decline autopsies, as documented in North American and European studies [10–13].

In France, the 2014 recommendations of the French National College of Gynecologists and Obstetricians (CNGOF) specified a minimum set of investigations for the postmortem examination after a fetal death when the family provides consent [14] and the French Health Authority also published a protocol for a postmortem exam after a fetal or neonatal death [15]. However, there are no recent national data on the autopsy rate, although the National Academy of Medicine reported in 2015 that fetal and neonatal autopsies were more often accepted than those for adults or children [16]. The limited data that do exist in the literature document a variable autopsy rate, from 87 to 45%, although parental refusals appear to be increasing in more recent years [17–19].

In this study, we aimed to describe the autopsy acceptance rate and to understand the factors associated with declining an autopsy after stillbirth in a disadvantaged French district with a high migrant population. Between 1992 and 2015, the costs of autopsies for fetal and neonatal deaths in this district were completely covered by the local council (Conseil Général) and there was therefore no financial burden associated with an autopsy for families or for hospitals [20]. We used a mixed-method approach using data from medical records and interviews with women experiencing a stillbirth collected as part of a population-based audit in the district in 2014.

## Methods

### Study design

This was a mixed methods study using data from an audit of all stillbirths and neonatal deaths occurring in the 11 maternity hospitals of the French district of Seine-Saint-Denis in 2014 and in-depth interviews with mothers who experienced a perinatal death. The study was funded by the Ile-de-France Regional Health Agency and aimed to understand the higher rates of perinatal mortality in this district compared to the other districts of the Ile-de-France region.

The study included all women experiencing a stillbirth, from 22 weeks of gestation, or a neonatal death who delivered in a maternity unit in Seine-Saint-Denis in 2014 and who provided consent to participate. A contact person for the study, midwife or nurse manager was designated among health personnel in each hospital and contacted the coordination team to report cases of stillbirth or neonatal death. This person also provided information to families about the study. Every three months, inclusions were verified against birth registers and neonatal admission registers by study investigators. During the postpartum stay in the hospital, the contact person invited women to participate in an in-depth interview with a research midwife 2 to 4 weeks after discharge from the maternity unit and collected contact information for those who agreed. Out of 249 eligible women, 218 consented to participate. Refusals were concentrated in the first two months of the study due to difficulties in the protocol that were corrected.

### Study population

For this analysis, we included the 156 stillbirths in the study (out of 172 eligible stillbirths over the study period), corresponding to 151 women. Fifty-four women accepted an interview.

### Data collection

Maternal demographic, socioeconomic and clinical characteristics as well as health care received during pregnancy and delivery were abstracted from medical records available in the maternity and neonatal units using a detailed, structured data collection instrument by three experienced midwives who received additional training for the study. The investigators also recorded whether an autopsy was proposed and its acceptance by the parents (Yes, no, information not noted).

For the maternal interviews, an interview guide was developed with pre-established questions on maternal characteristics and health care provided during pregnancy, delivery and after the stillbirth or neonatal death. The interview guide included a question about whether an autopsy was proposed by the medical team, if the parents accepted the autopsy (yes, no, do not wish to respond) and for both negative and positive responses, the reasons for their decision. The interview guide was developed with psychologists experienced in bereavement care as well as a sociologist and an anthropologist with expertise in qualitative data collection. The women were contacted 2 to 4 weeks after discharge to set up an appointment for an interview. This time frame was chosen in consultation with the psychologists in order to avoid the period of initial shock from the loss and to contact women when the experience was still relatively recent and before they returned to work. Interviews could be conducted in the woman’s home, a health care center that made space available for the project, or other locations (park, café), depending on the woman’s wishes. For women/couples who did not speak French well, translators with previous experience in qualitative research accompanied the investigators (5 interviews). The midwives of the research team received training in qualitative data collection methods and they took detailed notes during the interviews. They then transcribed their notes and other observations as soon as possible after the interview. The decision was made not to record the interviews because of concern about a higher rate of refusal. The interviews lasted a mean duration of 90 min, with a range from 40 min to 4 h.

### Analyses

We first described the sample of women experiencing a stillbirth and compared women with and without an interview. We then compared demographic and clinical characteristics between women accepting and refusing an autopsy using data on all women experiencing a stillbirth. For this analysis, we included variables available from the medical chart review that we hypothesized might affect the decision to accept an autopsy based on the previous literature: maternal age, parity, country of birth, insurance status, marital status, need for an interpreter, obstetrical history, body mass index and smoking. We used chi square or Fisher’s exact tests to compare across groups.

For the analyses of the maternal interviews, free text responses about the autopsy were extracted by one investigator (PS) using N’Vivo 10 QSR to facilitate the coding. Thematic analysis was then carried out. Once citations were selected for the manuscript, they were translated into English by a professional translator.

#### Ethical authorizations

This study was approved by Inserm’s ethical committee (IRB00003888), the French Advisory Committee on Use of Health Data in Medical Research and the French National Commission for Data Protection and Liberties.

## Results

### Characteristics of the women included in the audit

Twenty-six percent of women included in the audit were 35 years and over, 38% were having their first child and 7% had a multiple pregnancy. Over 60% were born outside of France and 18% were not living in a couple, 32% did not have health insurance under regular French insurance programs and 15% had a language barrier noted in medical records.

Women who accepted the interviews were similar to those who refused with respect to their age, parity, country of birth and nationality (Table 1). However, women who accepted the interviews were less likely to have no health insurance coverage (2% vs. 11.3%), although proportions with emergency or government provided universal insurance were similar. Medical and obstetric history and complications of the current pregnancy were also similar.

**Table 1:** Characteristics of the sample by whether the mother accepted an interview

	Without interview	%	With Interview	%	*P* value*
	*N* = 97		*N* = 54		
Age n(%)					
< 25 years	18	18.8	8	14.8	.686
25–35 years	55	57.3	30	55.6	
≥ 35 years	23	24.0	16	29.6	
Parity n(%)					
Nullipare	36	37.5	21	38.9	.867
Multipare	60	62.5	33	61.1	
Multiple birth					
No	90	92.8	50	92.6	.965
Yes	7	7.2	4	7.4	
Country of birth n(%)					
France	29	35.4	24	44.4	.487
Other Europe	5	6.1	1	1.9	
Northern Africa	12	14.6	10	18.5	
Sub-Saharan Africa	21	25.6	13	24.1	
Other countries	15	18.3	6	11.1	
Level of education					
No schooling/primary only			4	7.4	
Lower secondary			10	18.5	
Higher secondary			20	40.0	
Tertiary			20	37.0	
Insurance coverage					
French social security	50	62.5	38	77.6	.096
Gvt subsidised/emergency	21	26.3	10	20.4	
None	9	11.3	1	2.0	
Family situation					
In a couple	72	82.8	41	80.4	.728
Single mother	15	17.2	10	19.6	
Language barrier in medical records					
Language barrier not mentioned	80	83.3	47	88.7	.379
Noted	16	16.7	6	11.3	
Smoking during pregnancy					
No	75	87.2	46	95.8	.135
Yes	11	12.8	2	4.2	
Body Mass Index					
Underweight (< 18.5)	3	3.6	1	2.2	.811
Normal weight (18.5–24.9)	42	50.6	20	43.5	
Overweight (24–29.9)	20	25.0	13	28.9	
Obese (30+)	18	21.7	12	26.1	
Previous miscarriage^a^	19	19.6	17	11.3	.114
Previous stillbirth^a^	6	6.2	2	3.7	.712
Pregnancy complications^a^					
Hypertensive disorders	12	13.2	7	13.2	.997
Fetal growth restriction	17	17.5	6	11.1	.598
Preterm rupture of membranes	13	14.0	9	16.7	.660
Preterm Labor	16	16.8	12	22.2	.419

NOTE: *Chi Square or Fisher’s exact test.

^a^denominator varies because of some missing data

According to notes in the medical records, autopsies were proposed to 96.7% of women who experienced a stillbirth as described in Table 2. The information in the medical records was confirmed by all the women who were interviewed. Autopsies were proposed by obstetricians or midwives in the maternity hospital or doctors and nurses in the neonatal unit. Only one hospital had a fetopathologist (MB). She was involved in proposing autopsies in this hospital.

**Table 2:** Autopsies proposed and carried out for stillbirths in the District of Seine-Saint-Denis in 2014

	Total	Autopsy proposed (as % of total)		Autopsy carried out (as % of total)	
	*N*	*n*	%	*N*	%
Stillbirths	156	150	96.2	59	37.8
Women delivering stillbirths	151	146	96.7	59	39.1
Women with interviews	54	53	98.1	17	31.5

### Autopsy acceptance and associated social and clinical characteristics

Over one-third of families, 39.1%, agreed to an autopsy. This proportion was slightly lower in the sub-population of women who were interviewed: 31.5%. As shown in Table 3, the autopsy rate was associated with parity (49.1% of nulliparous women versus 33.3% of multiparous women) and country of birth (39.6% of women born in France versus 18.2% of women born in Northern Africa). Other variables were not related to the probability of accepting an autopsy.

**Table 3:** Autopsy rate by maternal and pregnancy characteristics

	Total Women *N* = 151	Autopsy Accepted (*n*)	Autopsy accepted (%)	*P* value^*^
Age *n*(%)				
< 25 years	26	13	50.0	0.464
25–35 years	85	32	37.6	
≥ 35 years	39	14	35.9	
Parity n(%)				
Nullipare	57	28	49.1	0.055
Multipare	93	31	33.3	
Multiple birth				
No	140	57	40.7	0.203
Yes	11	2	18.2	
Country of birth *n*(%)				
France	53	21	39.6	0.045
Other Europe	6	6	100.0	
Northern Africa	22	4	18.2	
Sub-Saharan Africa	34	12	35.3	
Other countries	21	11	52.4	
Level of education				
No schooling/primary only	4	1	25.0	0.823
Lower secondary	10	3	30.0	
Higher secondary	20	5	25.0	
Tertiary	20	8	40.0	
Insurance coverage				
French social security	88	33	37.5	0.456
Gvt subsidised/emergency	31	9	29.0	
None	10	5	50.0	
Family situation				
In a couple	113	43	38.1	0.848
Single mother	25	9	36.0	
Language barrier in medical records				
Language barrier not mentioned	127	48	37.8	0.28
Noted	22	11	50.0	
Smoking during pregnancy				
No	121	45	37.2	0.527
Yes	13	6	46.2	
Body Mass Index				
Underweight (< 18.5)	4	1	25.0	0.951
Normal weight (18.5–24.9)	62	26	41.9	
Overweight (24–29.9)	33	13	39.4	
Obese (30+)	30	13	43.3	
Previous miscarriage	36	16	44.4	
Previous stillbirth	8	3	37.5	
Pregnancy complications				
Hypertensive disorders	19	6	31.6	
Fetal growth restriction	23	12	52.2	0.364^+^
Preterm rupture of membranes	22	7	31.8	0.486^+^
Preterm Labor	28	8	28.6	0.283^+^

NOTE *Chi Square or Fisher’s exact test. + compared to women without this complication

### Reasons given by women for accepting or declining an autopsy

The main themes found in the analysis of women’s responses about why they accepted or declined the autopsy were: medical reasons, explanation from healthcare providers and, only when the autopsy was declined, the invasive nature of the examination and religious prohibitions.

- Medical reasons

The reasons given by women who accepted an autopsy in this study focused on the benefits of having more information, particularly for future pregnancies: “I want to know what happened” *(Interview 45; Interview 55)*. “I have an appointment tomorrow with the doctor who did the autopsy. I want to know if I can have another child without risk” *(Interview 31)*. One woman mentioned the usefulness of autopsy results for other parents: “They assured me that I might have other children, and so it was necessary to know for the future and to help others, even though I wouldn’t wish that on anyone” *(Interview 34)*.

Women also provided medical reasons for their decision to decline the autopsy. “The cause was obvious, you saw the cord marks in her neck; it wasn’t worth it to make her suffer” *(Interview 54)*. “We know it was the umbilical cord” *(Interview 10)*.

- Explanations from healthcare providers

In several cases it seemed that the women understood from their discussions with medical personnel that the autopsy would not provide additional information and even seemed to be advised against accepting a postmortem examination: “The doctor came to see me saying they knew medically what had happened and why the baby died (placental abruption)” *(Interview 27)*; “My husband would have wanted to, but there, they said there were no problems for the baby, that everything was due to the placenta” (Interview 33). These reasons were given by women who had vascular and placental complications or umbilical cord complications that were considered to be responsible for the death.

For some women interviewed, the quality of the information provided to them was perceived in retrospect, to be insufficient: “Because we weren’t sure about the reason. The midwives didn’t give convincing arguments. I regret it, because the gynecologist explained it to me after” *(Interview 7)*. In one other case, the autopsy did not seem to be proposed or discussed with the family: “In fact, they didn’t really offer it to me. The doctor said: ‘We’re sending him to the pathologist.’ Then I said, No, it’s too painful to think they are going to cut up my baby. He’s already been dead for 10-15 days. I didn’t want them to hurt him. But we did an analysis of the placenta” *(Interview 58).*

In this study, only one woman mentioned that she had been convinced by the medical personnel: “I didn’t want it, but they told me it was necessary because he was so little” *(Interview 14)*.

- The nature of the investigation

The invasive nature of the investigation was brought up by women to explain why they declined the autopsy: “When they explained what they would do, it made my heart ache.” *(Interview 37);* “So he wouldn’t be hurt anymore because he was so little” *(Interview 12*). Women expressed their concern about further harming the child and wanting to leave the child in peace: “I didn’t want them to touch him, to hurt him” *(Interview 8)* or “Opening up babies, that’s too hard” *(Interview 33)*.

- Religious reasons

Religious reasons were a final theme, either because women stated that there was explicit proscription of the procedure or because of the timing of burial requirements.: “Our religion doesn’t do them” *(Interview 52)*. In our study, most of the women bringing up religious concerns were Muslim, as they explained. However, religious proscription was often not the only reason or the first reason. For instance, a woman who had an induced delivery at 22 weeks of gestation for severe preeclampsia and fetal growth restriction very likely of vascular origin confided to the interviewer: “According to what they told me, the problem didn’t come from him; and then my religion doesn’t allow autopsies” *(Interview 50)*. In the medical file, the refusal of the autopsy is formulated differently: “Mother repulsed by the technique and not convinced of any contribution to diagnosis”. “They told me that there was very little chance that they’d know why, and then, for us, and for her father too, it’s forbidden.” *(Interview 35)*. Moreover, two women, one born in Senegal and the other in Mali were planning a burial in their home countries and were concerned that the autopsy could cause delays *(Interviews 4 and 12)*.

## Discussion

In our study in a disadvantaged French district, we found that autopsies were proposed to almost all women experiencing stillbirth in line with national and international recommendations. Parents accepted an autopsy in only 39.1% of all cases, despite full coverage of autopsy costs by the district authorities. Multiparous women and women born in Northern Africa were more likely to decline an autopsy. The reasons given by women for their decisions echo those found in previous studies. Women who accepted the autopsy primarily focused on getting knowledge about what happened, especially for future pregnancies but many refusals were related to a belief that meaningful information about the cause or the death would not be obtained from the autopsy. This appeared to reflect comments conveyed by the medical personnel when the examination was explained to the families. Concerns about the invasive nature of the examination were also widely expressed and women did not want to inflict anything more on their child. Religious reasons were also put forth to explain declining the autopsy, although this was rarely the only or even the first reason.

### Strengths and limitations

The strengths of this study include its prospective population-based design which made it possible to include almost all women experiencing a stillbirth in the district of Seine-Saint-Denis in 2014; one third accepted a semi-structured interview a few weeks after discharge from hospital, enabling us to ask women whether they had been offered an autopsy and about their reasons for accepting or declining the examination. We were able to compare the characteristics of the population of women accepting an interview with those that declined and found that their socioeconomic and medical characteristics were similar, although there were fewer women in extreme situations, i.e. without any insurance. Further, the fact that the study was not focused on the decision to accept an autopsy meant that acceptance of the interview was likely independent of opinions about autopsies. Finally, because the district covers the costs of autopsies, this study makes it possible to investigate women’s choices made in the absence of financial constraints. Limitations are the narrow scope of the questions about the autopsies due to the broader focus on the study. Also, since the interviews were not recorded, the quality of the verbatim quotes may have been affected by the transcription of the interviewers, although they were encouraged to take in-depth notes and to transcribe the interviews quickly after they had been conducted.

### Interpretation in light of previous research

Women accepting an autopsy were 39.1% of the study population, which is on the lower side of previous estimates of autopsy acceptance from studies in France [17, 19]. However, it appears that autopsy rates are declining. The most recent data from the REHOP stillbirth register in three departments confirm this perception with rates as low or lower as those found in Seine-Saint-Denis, ranging between 39.3 and 20.3% from 2013 to 2016 (personal communication, Dr. Anne Ego, director of the RHEOP registry). The districts participating in RHEOP are of average to high socioeconomic status. The autopsy acceptance rate in Seine-Saint-Denis is lower than those from other studies internationally. In the UK in 2014–2016, 49,4% of women experiencing a stillbirth accepted a post-mortem, with a small increase during the 3 years period [21] and a recent Australian study found an autopsy rate of 47% [22] .

Our results from the quantitative study as well as the qualitative analyses about the factors affecting the acceptance of an autopsy are concordant with those found previously [17, 19]. These studies report a wide range of reasons for refusal including negative perceptions of the procedure, compounded by the assumption that the autopsy will not provide a clear response about the cause of death [23], emotional distress and apprehension about a long wait for results [24], a perception of the invasiveness of the autopsy [25], insufficient or discouraging explanations from healthcare professionals [25, 26], concerns about the cultural or religious acceptability of a postmortem exam and funeral delays [27], high costs [23] and publicized scandals around retaining or reuse of organs, [11]. Because autopsy costs were covered by the district authorities, one theme emerging from previous research – the financial burden associated with autopsies – was not mentioned by the women in our study.

Women who accepted focused primarily on the importance of knowing the cause and implications for future pregnancies; this concern was reflected in the higher acceptance rates among women having their first child.

Our results illustrate the need to convince health professionals of the value of carrying out autopsies and developing policies to improve communication about the post-mortem examination’s benefits and utility. While autopsies were systematically proposed to families experiencing stillbirths, the way the examination was presented differed and appeared to have a strong impact on decisions. As shown by an example of discordance between reasons given in medical records and in the mother’s interview, some situations of misunderstanding between patients and medical staff may exist. But above all, analyses of the women’s comments showed that in many cases the medical personnel were not convinced themselves of the utility of the examination; especially when the stillbirth was associated with vascular or cord complications. However, a review of contemporary studies on the value of perinatal autopsy showed that the autopsy could reveal a previously undiscovered diagnosis, a change in the diagnosis or additional information in 22 to 76% of cases [12].

A Delphi consensus process with health professionals in the Seine-Saint-Denis district was conducted prior to this research on the reasons for the high rates of stillbirth. The panel agreed that religious reasons explained the low rates of prenatal screening and pregnancy termination in the district [28]. Information about religion cannot be asked in research in France without special authorization from ethical boards but some women explained their decision by referring to their Muslim religion, although this was not often given as the primary reason. Their comments suggest that medical staff may be hesitant to insist or provide arguments in favor of the examination for these women. It is noteworthy that there is no formal proscription of autopsies in any of the three monotheistic religions [29]. In Islam, however, the time between death and burial is short [30]. In France, parents choose whether to have a funeral for their stillborn baby, as this is not required by law. However, autopsies can be carried out quickly to allow a funeral within a short period. There seems to be a confusion between the time needed to carry out the autopsy and the time to get the final results, which depends on the analyses of laboratory tests. In a study by Heazell et al., the authors remark that «The majority of staff ranked workload, negative publicity, religion and cultural issues as important barriers, whereas most parents did not » [24]. Further, several studies have suggested that parents would have liked the staff to have been more proactive about explaining benefits of the examination to help them to overcome their initial negative reactions [25]. Research on implicit bias by social psychologists [31] or social loss by interactionist sociologists has shown how conceptions by caregivers about their patients’ preferences can have an impact on the care that they provide [32]. While we did not interview the medical personnel, their professional training and roles as well as cultural and religious views may affect their discussion of autopsies with families [33]. An English study with 792 obstetric health personnel (midwives, obstetricians and fetopathologists), showed that the role of the health professional, their previous training about autopsies as well as their emotional responses to the idea of an autopsy affected their practices. Midwives and young obstetricians were less likely to propose autopsies to families [9]. Other studies have confirmed these results [10, 26, 34]. Stock et al. showed that the rate of autopsy acceptance rose when a department of fetopathology opened in the maternity hospital – in part because of the possibility of having common staff meetings, but also because parents were reassured by having the fetopathologist explain the postmortem and its benefits [10].

Autopsies are important for research on the causes of stillbirth and for developing effective preventive policies. In the absence of an autopsy, a detailed examination of the placenta remains essential [35]. A focus on less invasive examinations and their contribution to establishing a diagnosis [36–40] would seem to correspond to the wishes of parents who decline an autopsy [27]. Other innovative solutions have also been suggested. An Indian team, for instance, proposed immediate autopsies in the delivery room by the doctors in the team in order to take samples to be sent to the laboratory, thereby allowing for a very rapid return of the body to the family. This study found higher acceptance by the family when they were assured of being able to respect culturally mandated funeral rites [41]. It is noteworthy, however, that a review of the literature on autopsies was not able to find a study evaluating interventions to support parental decisions about an autopsy [42].

## Conclusion

Our study found low autopsy rates in a disadvantaged high-migrant district in France despite full coverage of all costs by the district council and systematic proposal of the exam to women by health professionals. Interviewing women provides a better understanding of this finding. The reasons given for declining autopsy mirrored those from research carried out in a range of contexts. Most saliently, many women who declined believed that limited additional information would be gained from the autopsy, while those who accepted did so because wanted to understand more about the causes. The women’s statements suggested that many health professionals did not seem to be convinced about the benefits of the autopsy or were unable to explain them persuasively. It is possible that health professionals had their own beliefs about the women’s or families’ wishes which made them reluctant to more actively encourage acceptance. Our results thus illustrate the importance of including health professionals in research on this topic and of providing support for medical personnel, including training about the benefits of autopsies and how to present them to reluctant families or families with concerns about religious proscriptions and burial rites. Research on implicit biases and their impact on medical care would be relevant for understanding how caregivers’ perceptions affect counselling. Innovating with procedures to ensure accommodation of parents’ wishes for rapid burials or for less invasive methods may also help to overcome reticence felt by many parents.
